# An Affinity Propagation-Based DNA Motif Discovery Algorithm

**DOI:** 10.1155/2015/853461

**Published:** 2015-08-10

**Authors:** Chunxiao Sun, Hongwei Huo, Qiang Yu, Haitao Guo, Zhigang Sun

**Affiliations:** School of Computer Science and Technology, Xidian University, Xi'an 710071, China

## Abstract

The planted (*l*, *d*) motif search (PMS) is one of the fundamental problems in bioinformatics, which plays an important role in locating transcription factor binding sites (TFBSs) in DNA sequences. Nowadays, identifying weak motifs and reducing the effect of local optimum are still important but challenging tasks for motif discovery. To solve the tasks, we propose a new algorithm, APMotif, which first applies the Affinity Propagation (AP) clustering in DNA sequences to produce informative and good candidate motifs and then employs Expectation Maximization (EM) refinement to obtain the optimal motifs from the candidate motifs. Experimental results both on simulated data sets and real biological data sets show that APMotif usually outperforms four other widely used algorithms in terms of high prediction accuracy.

## 1. Introduction

Transcription factor binding sites (TFBSs) are short and conserved nucleotide fragments (usually ≤ 30 bps) in the cis-regulatory regions of genes in DNA sequences. They interact with transcription factors (TFs) and affect the gene expression. Identification of TFBSs, that is, motif discovery [1], is a fundamental problem for its importance to understand the structure and function of gene expression.

In this paper, we focus on the planted (*l*, *d*) motif search (PMS) problem [2], a widely accepted formulation of motif discovery problem. Given a set of input* n*-length DNA sequences *X* = {*X*_1_, *X*_2_,…, *X*_*t*_} and two nonnegative integers *l* and *d*, the aim of the PMS is to find an* l*-mer *M* (an* l*-length string), which occurs in each of the *t* sequences with up to *d* mutations. The* l*-mer *M* is called a (*l*, *d*) motif and each mutation of *M* is called a motif instance.

The existing algorithms to solve PMS problem include two main categories. One is exact algorithms, most of which use consensus sequences [3] to represent motifs. The exact algorithms are guaranteed to obtain the optimal motif. Recently, the research of exact algorithms mainly concentrates on pattern-driven algorithms. All the* l*-length string patterns are taken as candidate motifs, and the string patterns occurring in all input sequences with up to *d* mutations are the motifs. Typical pattern-driven algorithms use various means to reduce time complexity [4–10]. PairMotif [4] selects multiple pairs of* l*-mer with relatively large distance from the input sequences to restrict the search space. Compared with recently proposed algorithms, PairMotif requires less storage space and runs faster on most PMS problems. PMS5 [7] computes the common* d*-neighbors of three* l*-mers using integer programming formulation, which is an efficient algorithm for solving the difficult instances of PMS: (21, 8) and (23, 9). Some other pattern-driven algorithms index the input sequences with a suffix tree to speed up the search of candidate motifs [11–14]. RISOTTO [11] is the fastest algorithm in the family of suffix tree algorithms for PMS problem and can solve the instance (15, 5) in 100 minutes. The initial search space of pattern-driven algorithms is *O*(4^*l*^). Therefore, pattern-driven algorithms are feasible for small motif length *l*  (*l* ≤ 20), but they will take long running time or have high space requirement with the increase of the motif length.

The other category is approximate algorithms, which commonly use position weight matrixes (PWMs) [15] to represent motifs. They can report results in a short time but often get trapped in local optimal solutions. Most approximate algorithms attempt to maximize the score function of how likely a subsequence of an input sequence is a motif instance, using statistical analysis [16–23]. MEME [18] and Gibbs sampling [20] are well-known approximate algorithms. MEME finds motifs by optimizing the PWMs using the Expectation Maximization (EM). Based on MEME, there are some extension algorithms like Projection [21] and MCEMDA [22]. Projection projects all* l*-mers from the input sequences onto many buckets by hashing and then derives the consensus sequences to select some valid buckets. After the effective initialization step, EM algorithm is used for refinement. MCEMDA is a modification of the EM algorithm in that the expectation in the E-step is computed numerically through Monte Carlo simulation. Gibbs sampling is a Markov Chain Monte Carlo (MCMC) approach. Based on Gibbs sampling strategy, there are some modifications that have also been described [24, 25]. One that stands out is AlignACE [25], which is a Gibbs sampling algorithm for identifying the overrepresented motifs in a set of DNA sequences. Furthermore, some graph-theoretic methods either based on clustering or on heuristic search have also been introduced in the field of motif discovery [26–28]. CRMD [26] uses an entropy-based clustering to find good starting candidate motifs from the input sequences and then employs an effective greedy refinement to search for optimal motifs from the candidate motifs. VINE [28] is a graph clustering algorithm for motif discovery by finding *t*-cliques in a *t*-graph in polynomial time. Generally, the approximate algorithm has speedy runtime and minimal memory consumption. Sometimes, however, they cannot converge to the global optimal.

In this paper, we propose a new algorithm, APMotif, to solve motif discovery problem. APMotif first applies Affinity Propagation (AP) [29] clustering in DNA sequences to find highly conserved candidate motifs. APMotif then employs an effective EM refinement to search for optimal motifs from the candidate motifs. Experimental results show that APMotif has competitive prediction accuracy compared to that of previously developed algorithms.

## 2. Materials and Method

Here, we first briefly describe the original Affinity Propagation clustering and Expectation Maximization algorithms used in the remainder of the paper. We then construct the similarity matrix for motif discovery. Finally, we describe the APMotif algorithm.

### 2.1. Affinity Propagation (AP)

Compared with other clustering approaches, AP clustering is an effective and fast clustering algorithm, especially for large data sets. Given a set of data points *X* = {*X*_1_, *X*_2_,…, *X*_*t*_}, AP clustering takes as input a collection of real valued similarities *s*(*i*, *k*) between the pairs *X*_*i*_ and *X*_*k*_,  *i*, *k* ∈ {1,2,…, *t*}. According to the similarities between data points, AP clustering recursively calculates two types of messages: the responsibility *r*(*i*, *k*), reflecting the suitability of point *X*_*k*_ as the exemplar for point *X*_*i*_, and the availability *a*(*i*, *k*), indicating how appropriate it would be for point *X*_*i*_ to choose point *X*_*k*_ as its exemplar:

(1) ri,k=si,k−maxXk′≠Xk⁡ai,k′+si,k′,ai,k=min⁡0,rk,k+∑Xi′≠Xi,Xkmax⁡0,ri′,kif  Xi≠Xk,ak,k=∑Xi′≠Xkmax⁡0,ri′,k.

Upon convergence, AP clustering selects a subset of data points as exemplar and assigns every nonexemplar point to exactly one exemplar. The exemplar *e*(*i*) = *X*_*k*_ associated with point *X*_*i*_ is finally defined as follows:

(2) $$\begin{array}{c} e\left(\;i\;\right)=arg\;\underset{X_{k}}{\max}\left\{\;r\left(\;i,k\;\right)+a\left(\;i,k\;\right)\;\right\}. \end{array}$$

The AP clustering is terminated when the exemplar remains unchanged for a user-set number of iterations.

### 2.2. Expectation Maximization (EM)

For EM algorithm, given the DNA sequences *X* = {*X*_1_, *X*_2_,…, *X*_*t*_}, each sequence consists of two components which model the motif and nonmotif (“background”) positions in the sequence. The starting positions of the motif in each sequence are unknown and represented by the variables (“missing data”) *Z* = {*Z*_*i*,*j*_∣1 ≤ *i* ≤ *t*, 1 ≤ *j* ≤ *n* − *l* + 1}, where *Z*_*i*,*j*_ = 1 if a motif starts at position *j* in the sequence *X*_*i*_, and *Z*_*i*,*j*_ = 0 otherwise.

EM algorithm attempts to maximize the expectation of the logarithm of the joint likelihood of the model.

The main procedure of EM algorithm repeats iteratively the following two steps:

(3) $$\begin{array}{c} E\text{-}step\text{:}\;Z^{\left(T\right)}=\underset{\left(Z\;|\;X,\theta^{\left(T\right)}\right)}{E}\left[Z\right], \end{array}$$

(4) $$\begin{array}{c} \text{M-step:}\;\theta^{\left(T+1\right)}=\underset{\theta }{arg\;max}\underset{\left(Z\;|\;X,\theta^{\left(T\right)}\right)}{E}\left[\log p\left(X,Z\;|\;\theta \right)\right]. \end{array}$$

In (4), the logarithm of the joint likelihood of the model is defined as follows:

(5) $$\begin{array}{c} \log p\left(\;X,Z\;|\;\theta \;\right)=\overset{t}{\underset{i=1}{\sum }}\overset{n-l+1}{\underset{j=1}{\sum }}Z_{i,j}\log p\left(\;X_{i}\;|\;Z_{i,j}=1,\theta \;\right), \end{array}$$

where

(6) $$\begin{array}{c} \theta =\left[\;\theta_{0},\theta_{1}\;\right]=\left[\;P_{0},P_{1},P_{2},\ldots ,P_{l}\;\right]={\left[\;P_{w,m}\;\right]}_{4\times \left(l+1\right)} \end{array}$$

is the vector containing all the parameters of the model and *P*_*w*,*m*_ is the probability of the character *w* ∈ {*A*, *T*, *C*, *G*} occurring at either a background position (*m* = 0) or a motif position (1 ≤ *m* ≤ *l*).

In (5), the conditional probability for a sequence containing a motif is defined as follows:

(7) log⁡pXi ∣ Zi,j=1,θ=∑k=0l−1Ii,j+kTlog⁡Pk+∑k∈Δi,jIi,kTlog⁡P0,

where *I*(*i*, *j*) indicates a vector whose entries are all zeros except the one corresponding to the character at position *j* in the sequence *X*_*i*_. Δ_*i*,*j*_ is the set of positions of the background in the sequence *X*_*i*_.

### 2.3. Construction of Similarity Matrix for Motif Discovery

In the original AP clustering, given two random* l*-mers *x*_*i*_ and *x*_*k*_ from *t* DNA sequences *X* = {*X*_1_, *X*_2_,…, *X*_*t*_}, the similarity is set as the negative Hamming distance between* l*-mers *x*_*i*_ and *x*_*k*_; that is, *s*(*i*, *k*) = −*d*_*H*_(*x*_*i*_, *x*_*k*_) [29], which cannot describe the property of DNA sequences clustering effectively. According to the feature of PMS that two motif instances of the same motif cannot differ by more than 2*d* positions, and the maximum similarity principle, we employ pairwise constraints and variable-similarity measure [30] to modify the similarity as follows:

(8) $$\begin{array}{c} s\left(\;i,k\;\right)=-\rho \times d_{H}\left(\;x_{i},x_{k}\;\right)\times L\left(\;x_{i},x_{k},X\;\right), \end{array}$$

where

(9) ρ=R1if  dHxi,xk∈0,dR2if  dHxi,xk∈d,2d+∞if  dHxi,xk∈2d,4d,Lxi,xk,X=+∞if  xi ∈l Xp,  xk ∈l Xq,  p=q1otherwise.

*R*_1_∈ (1, +∞), *R*_2_∈ (0, 1], and *x*_*i*_ ∈_*l*_ *X*_*p*_ denotes *x*_*i*_ is an* l*-mer of the sequence *X*_*p*_.

Based on the similarity in (8), the similarity between data points is more accurate and only tiny subsets of the data points are required to exchange messages, so AP clustering can not only increase clustering accuracy but also decrease runtime. Its theoretical analyses are shown in Section 3.1.

**Figure 1:**
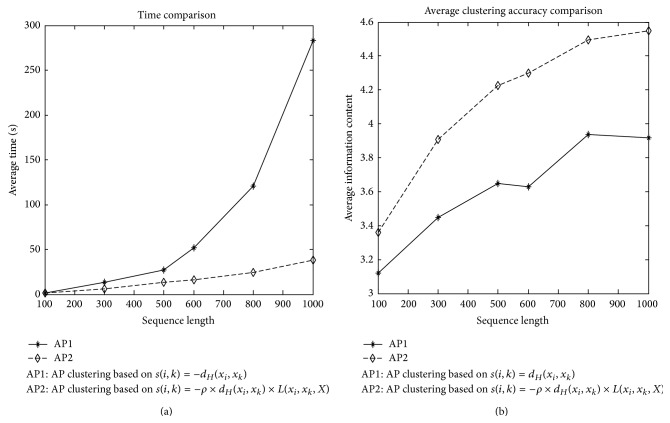
AP clustering results.

According to the two similarities: *s*(*i*, *k*) = −*d*_*H*_(*x*_*i*_, *x*_*k*_) and *s*(*i*, *k*) = −*ρ* × *d*_*H*_(*x*_*i*_, *x*_*k*_) × *L*(*x*_*i*_, *x*_*k*_, *X*), take the PMS instance (15, 4) with 20 sequences of different length between 100 and 1000 as an example; we show the comparison of runtime and clustering accuracy in Figure 1.

### 2.4. APMotif Algorithm

Under the assumption of exactly one occurrence of motif instance per sequence (OOPS) [1], to find the motif instances from the input DNA sequences *X* = {*X*_1_, *X*_2_,…, *X*_*t*_}, APMotif algorithm consists of the following stages:

(1) *Constructing Clusters*. Select the sequence *X*_1_ as the reference sequence, for each* l*-mer *x*_*k*_  (*k* = 1,2,…, *n* − *l* + 1) in *X*_1_ (reference subsequence), and construct cluster *C*(*x*_*k*_, *X*), which is the set composed by all the* l*-mer *x*′ in *X* − {*X*_1_} that *d*_*H*_(*x*_*k*_, *x*′) ≤ 2*d* and the* l*-mer *x*_*k*_.
(2) *Extracting Clusters*. For each cluster *C*(*x*_*k*_, *X*), use AP clustering and a filtering rule to generate a highly conserved cluster *C*′(*x*_*k*1_, *X*).
(3) *Refining Clusters*. For each filtered cluster *C*′(*x*_*k*1_, *X*), use EM refinement to obtain the distribution *θ*_*k*1_ and the objective function *Q*_*k*1_ of each cluster *C*′(*x*_*k*1_, *X*).
(4) *Verifying Motif Instances*. With the maximum distribution *θ*_max_ and the maximum objective function *Q*_max_, the* l*-mer *y* having the maximum log-likelihood log⁡*p*(*y*∣*θ*_max_) in each sequence is verified as a motif instance.

Based on the four stages, the APMotif algorithm is presented as in Algorithm 1.

In line (1), the set of the (*l*, *d*) motif instances *M* is initialized to an empty set. Lines (2)-(3) show the stage of constructing clusters. Lines (4)-(5) show the stage of extracting clusters. Lines (6)-(7) show the stage of refining clusters. Lines (8)–(12) show the stage of verifying motif instances. APMotif can discover the (*l*, *d*) motif instances in high prediction accuracy and output them in line (13).

Next, we explain each stage in detail.

**Stage 1 (construct clusters).**

The construction of clusters keeps the following simple observation that the Hamming distance between two motif instances of the same motif must be less than or equal to 2*d*. Generally, we choose the first sequence *X*_1_ as the reference sequence. As we do not know in advance which* l*-mer *x*_*k*_ in *X*_1_ is the motif instance, all the* l*-mers *x*_*k*_  (*k* = 1,2,…, *n* − *l* + 1) in *X*_1_ are regarded as the reference subsequences. Given an* l*-mer *x*_*k*_ in *X*_1_, the selected* l*-mers *x*′ in other sequence *X*_*i*_  (*i* = 2,…, *n* − *l* + 1) should satisfy *d*_*H*_(*x*_*k*_, *x*′) ≤ 2*d*, denoted as *B*(*x*_*k*_, *X*_*i*_) = {*x*′ : *x*′ ∈_*l*_ *X*_*i*_, *d*_*H*_(*x*_*k*_, *x*′) ≤ 2*d*}, where *x*′ ∈_*l*_ *X*_*i*_ denotes that *x*′ is an* l*-mer of *X*_*i*_. The cluster corresponding to the reference subsequence *x*_*k*_ is denoted as

(10) $$\begin{array}{c} C\left(\;x_{k},X\;\right)=\left\{\;x_{k}\;\right\}\cup \overset{n-l+1}{\underset{i=2}{⋃}}B\left(\;x_{k},X_{i}\;\right). \end{array}$$

The average number of* l*-mers in the cluster *C*(*x*_*k*_, *X*) is *p*_2*d*_ × *t* × (*n* − *l* + 1), where

(11) $$\begin{array}{c} p_{2d}=\overset{2d}{\underset{i=0}{\sum }}\left(\begin{array}{c} l\\ i \end{array}\right){\left(\;\frac{3}{4}\;\right)}^{i}{\left(\;\frac{1}{4}\;\right)}^{l-i} \end{array}$$

is the probability that the Hamming distance between two random* l*-mers is at most 2*d*.

**Stage 2 (extract clusters).**

For each cluster subset *C*(*x*_*k*_, *X*), we use the AP clustering to produce the high conserved cluster *C*′(*x*_*k*_, *X*) that contains the reference subsequence *x*_*k*_. If one of the reference subsequences *x*_*k*_  (*k* = 1,2,…, *n* − *l* + 1) is a motif instance, the corresponding cluster *C*′(*x*_*k*_, *X*) may be the true motif model.

For each cluster *C*′(*x*_*k*_, *X*), two types of metrics, information content (IC) and complexity scores [31], are employed to assess the quality of the cluster. The information content of the cluster *C*′(*x*_*k*_, *X*) is defined as

(12) $$\begin{array}{c} Q_{k}=IC\left(\;C^{\prime }\left(\;x_{k},X\;\right)\;\right)=\overset{l}{\underset{m=1}{\sum }}\;\overset{4}{\underset{w=1}{\sum }}p_{w,m}\log\frac{p_{w,m}}{p_{w,0}}, \end{array}$$

where *p*_*w*,*m*_ represents the probability of each character *w* ∈ {*A*, *T*, *C*, *G*} appearing at the position *m* of the* l*-mer, and where *p*_*w*,0_ is the background probability of character *w*. A higher IC value indicates a stronger potential of a cluster to be the true motif model.

The complexity score of the cluster *C*′(*x*_*k*_, *X*) is defined as

(13) $$\begin{array}{c} J\left(\;C^{\prime }\left(\;x_{k},X\;\right)\;\right)={\left(\;\frac{1}{4}\;\right)}^{l}\overset{4}{\underset{w=1}{\prod }}{\left(\;\frac{l}{\sum_{m=1}^{l}p_{w,m}}\;\right)}^{\sum_{m=1}^{l}p_{w,m}}. \end{array}$$

Note that the IC value cannot completely reflect the conservation of the motif model. The reason is that many noninformative repeated* l*-mers may lead to a higher IC value. Fortunately, these false positive clusters have lower complexity scores and they can be effectively filtered out.

Taking these into account, we propose the following rule to filter out some unqualified clusters.

*Rule.* If *J*(*C*′(*x*_*k*1_, *X*))>(1/(*n* − *l* + 1))∑_*k*=1_^*n*−*l*+1^ *J*(*C*′(*x*_*k*_, *X*)) and IC(*C*′(*x*_*k*1_, *X*))  >  (1/(*n* − *l* + 1))∑_*k*=1_^*n*−*l*+1^IC(*C*′(*x*_*k*_, *X*)), the cluster *C*′(*x*_*k*1_, *X*) will be stored as a candidate motif model.

According to the rule, a few clusters *C*′(*x*_*k*1_, *X*) with high IC value and high complexity scores are stored for EM refinement in Stage 3.

**Stage 3 (refine clusters).**

It is important to note that AP clustering is primarily an initialization strategy that produces starting points for EM refinement. Taking each cluster *C*′(*x*_*k*1_, *X*) as a starting point, we use the modified EM refinement to search for a motif model.

The E-step of EM calculates the expected value of the missing information *Z*_*i*,*j*_, which is the probability that a motif starts in position *j* of sequence *X*_*i*_.

*E-Step.* Consider

(14) $$\begin{array}{c} Z_{i,j}^{\left(T\right)}=\frac{p\left(X_{i}\;|\;z_{i,j}=1,\theta^{\left(T\right)}\right)}{\sum_{j=1}^{n-l+1}p\left(X_{i}\;|\;z_{i,j}=1,\theta^{\left(T\right)}\right)}. \end{array}$$

The M-step of EM reestimates distribution *θ* by maximizing the expected log-likelihood.

*M-Step*. Consider

(15) pw,mT=cw,m+ξm∑w∈Ωcw,m+ξmw∈Ω=A,T,C,G,cw,m=∑i=1t∑j=1n−l+1zi,jTIi,j+k−1,cw,0=cw−∑m=1lcw,m,

where *ξ*_*m*_ is the pseudocount to deal with the zero frequencies and *c*_*w*_ is the total number of the character *w* in all sequence *X*.

The EM algorithm is terminated when the object function *Q*, that is, Information Content, remains unchanged. After EM refinement, we can obtain the distribution *θ*_*k*1_ and the objective function *Q*_*k*1_ of each cluster *C*′(*x*_*k*1_, *X*).

**Stage 4 (select motif instances).**

Comparing each distribution *θ*_*k*1_, we find the maximum one *θ*_max_. For the distribution *θ*_max_, an* l*-mer *y* in one sequence with the maximum log-likelihood that is considered as a candidate motif instance:

(16) $$\begin{array}{c} \log p\left(\;y\;|\;\theta_{max}\;\right)=\max\overset{l}{\underset{m=1}{\sum }}\log p_{w,m}. \end{array}$$

Meanwhile, a candidate motif instance *y* should satisfy *d*_*H*_(*y*, *x*_motif_) ≤ *d*, where *x*_motif_ is the motif by using *θ*_max_ as the consensus.

Thus, the* l*-mer *y* that has the maximum log-likelihood under the distribution *θ*_max_ and satisfies *d*_*H*_(*y*, *x*_motif_) ≤ *d* is stored in the set of motif instances *M*.

## 3. Results and Discussion

Here, we first theoretically analyze the probability of *s*(*i*, *k*) = −*∞* and give its formula. We then show the experimental results of APMotif both on simulated data sets and real biological data sets.

### 3.1. Analysis of Similarity Matrix

It has been pointed out in [29] that the sparsity of the similarity matrix will lead to fast calculation since the information propagation needs not be performed if *s*(*i*, *k*) = −*∞*.

Given two random* l*-mers *x*_*i*_ and *x*_*k*_, coming from different sequences, which differ from the same* l*-mer *x*_0_ with up to 2*d* positions, the distance relationships between *x*_*i*_, *x*_*k*_, and *x*_0_ satisfy 0 ≤ *d*_*H*_(*x*_0_, *x*_*i*_) ≤ 2*d* and 0 ≤ *d*_*H*_(*x*_0_, *x*_*k*_) ≤ 2*d*. Let *p*(*α*, *β*) represent the probability of *d*_*H*_(*x*_0_, *x*_*i*_) = *α* and *d*_*H*_(*x*_0_, *x*_*k*_) = *β* corresponding to a sample space *Ω* = {〈*α*, *β*〉 : 0 ≤ *α* ≤ 2*d*, 0 ≤ *β* ≤ 2*d*}. Because *d*_*H*_(*x*_0_, *x*_*i*_) = *α* and *d*_*H*_(*x*_0_, *x*_*k*_) = *β* are independent of each other, *p*(*α*, *β*) can be calculated as follows:

(17) pα,β=pdHx0,xi=α,dHx0,xk=β=pdHx0,xi=α×pdHx0,xk=β,

(18) pdHx0,xi=α=2dα3α42d,

Let *p*(*d*_*H*_(*x*_*i*_, *x*_*k*_) > 2*d*) represent the probability that the Hamming distance between two random* l*-mers *x*_*i*_ and *x*_*k*_ is more than 2*d*.

Using Theorem of Total Probability, we have

(19) pdHxi,xk>2d=pdHxi,xk>2d ∣ dHx0,xi=α,dHx0,xk=β×pα,β,

where *p*(*d*_*H*_(*x*_*i*_, *x*_*k*_) > 2*d*∣*d*_*H*_(*x*_0_, *x*_*i*_) = *α*, *d*_*H*_(*x*_0_, *x*_*k*_) = *β*) represents the conditional probability of *d*_*H*_(*x*_*i*_, *x*_*k*_) > 2*d* given *d*_*H*_(*x*_0_, *x*_*i*_) = *α* and *d*_*H*_(*x*_0_, *x*_*k*_) = *β*.

Next, we discuss how to calculate the conditional probability *p*(*d*_*H*_(*x*_*i*_, *x*_*k*_) > 2*d*∣*d*_*H*_(*x*_0_, *x*_*i*_) = *α*, *d*_*H*_(*x*_0_, *x*_*k*_) = *β*).

According to *d*_*H*_(*x*_*i*_, *x*_*k*_) ≤ *d*_*H*_(*x*_0_, *x*_*i*_) + *d*_*H*_(*x*_0_, *x*_*k*_) and *d*_*H*_(*x*_*i*_, *x*_*k*_) > 2*d*, we can obtain

(20) $$\begin{array}{c} d_{H}\left(\;x_{0},x_{i}\;\right)+d_{H}\left(\;x_{0},x_{k}\;\right)>2d. \end{array}$$

For 0 ≤ *d*_*H*_(*x*_0_, *x*_*i*_) ≤ 2*d* and 0 ≤ *d*_*H*_(*x*_0_, *x*_*k*_) ≤ 2*d*, (20) can be written as

(21) dHx0,xi+dHx0,xk=2d+1+cc=0,1,…,2d−1.

Given *d*, for each *c*, we can find all the 2-tuple 〈*d*_*H*_(*x*_0_, *x*_*i*_), *d*_*H*_(*x*_0_, *x*_*k*_)〉 = 〈*α*, *β*〉 that satisfy (21).

Given *c*, for each 2-tuple 〈*α*_0_, *β*_0_〉, the conditional probability of *d*_*H*_(*x*_*i*_, *x*_*k*_) > 2*d* can be calculated as follows:

(22) pdHxi,xk>2d ∣ dHx0,xi=α0,dHx0,xk=β0=∑i=0cα0il−α0β0−i×3β0lβ0×3β0.

Considering all the values *c* = 0,1,…, 2*d* − 1 and all the 2-tuple 〈*α*, *β*〉, we calculate the conditional probability of *d*_*H*_(*x*_*i*_, *x*_*k*_) > 2*d* as follows:

(23) pdHxi,xk>2d ∣ dHx0,xi=α,dHx0,xk=β=∑c=02d−1∑α,β∑i=0cαil−αβ−i×3βlβ×3β.

According to (18) and (23), we can obtain

(24) pdHxi,xk>2d=∑c=02d−1∑α,β∑i=0cαil−αβ−i×3βlβ×3β×2dα3α42d×2dβ3β42d.

The probability *p*(*d*_*H*_(*x*_*i*_, *x*_*k*_) > 2*d*) is also the probability of *s*(*i*, *k*) = −*∞* corresponding to the condition that *d*_*H*_(*x*_*i*_, *x*_*k*_)∈(2*d*, 4*d*].

Meanwhile, when the two* l*-mers *x*_*i*_ and *x*_*k*_ are in the same sequence, the probability of *s*(*i*, *k*) = −*∞* in the similarity matrix is 1/(*t* − 1), where *t* is the sequence number.

For the (15, 4) problem instance with sequence number *t* = 20, the probability of *s*(*i*, *k*) = −*∞* obtained by (24) and *t* is 0.8405, when sequence length *n* varies from 100 to 1000.

In Table 1, by enumerating all the *s*(*i*, *k*) = −*∞* in the similarity matrix, the empirical result shows that the probability of *s*(*i*, *k*) = −*∞* accounts for more or less than 84% of the similarity matrix, which is consistent with the theoretical analysis.

### 3.2. Results on Synthetic Data Sets

We generate the synthetic data sets as follows: first, we generate a motif *M* of length *l* and *t* independent and identically distributed (i.i.d) sequences *X* of length *n*. Then, we implant (*l*, *d*) instance, which differs from the motif *M* with up to *d* positions, into a random position in each sequence.

The nucleotide level performance coefficient (nPC) defined by Pevzner and Sze [2] is used to evaluate the motif prediction accuracy:

(25) $$\begin{array}{c} nPC=\frac{\left|K\cap P\right|}{\left|K\cup P\right|}. \end{array}$$

*K* is the set of *l* × *t* base positions in the *t* known motif instances, and *P* is the corresponding set of *l* × *t* base positions in the *t* predicted motif instances. The value of nPC is between 0 and 1; the larger the value of nPC, the higher the accuracy of the predicted motif.

Table 2 shows the comparison of the mean nPC obtained by APMotif and four other representative algorithms: MEME [16–18], Gibbs sampling [20], Projection [21], and VINE [28]. For each of the (*l*, *d*) combinations, all the five algorithms are run once on each of 10 randomly generated sets of input sequences (*t* = 20, *n* = 600). APMotif constitutes a simple and effective method which groups the significant* l*-mers to form the optimal clusters so that the motif instances can be predicted with high accuracy. APMotif has the highest mean nPC on the instances (11, 3), (12, 3), (18, 6), and (19, 7), and the second highest mean nPC on the instances (15, 4), (16, 5), which proves that APMotif is relatively robust in various problem instances.

In Table 3, we compare the nPC of APMotif on problem instances with longer background sequences. Since the longer a sequence is, the more noisy* l*-mers will be yielded, this makes it difficult to discover the true motifs. We fix the (*l*, *d*) instance as (15, 4) instance, one of the most popular benchmarks for motif discovery problem, and vary the sequence length *n* from 100 to 1000. For each setting, 10 i.i.d data sets are generated, each containing 20 sequences. The nPC of APMotif is over 95% for sequences of various lengths between 100 and 1000, much greater than that of Projection, MEME, Gibbs sampling, and VINE. The reason why the performance of APMotif is stable over the sequence length is that APMotif has strong ability of filtering noisy* l*-mers. With each sequence length *n* increasing (*n* ≥ 600), APMotif still maintains its advantage in the prediction accuracy. For example, when the sequence length is 1000 bps, the nPC of Projection, MEME, VINE, and Gibbs sampling are 88%, 76%, 91%, and 8%, respectively, while APMotif algorithm shows its advantage with the nPC 95%.

### 3.3. Results on Real Biological Data Sets

**Figure 2:**
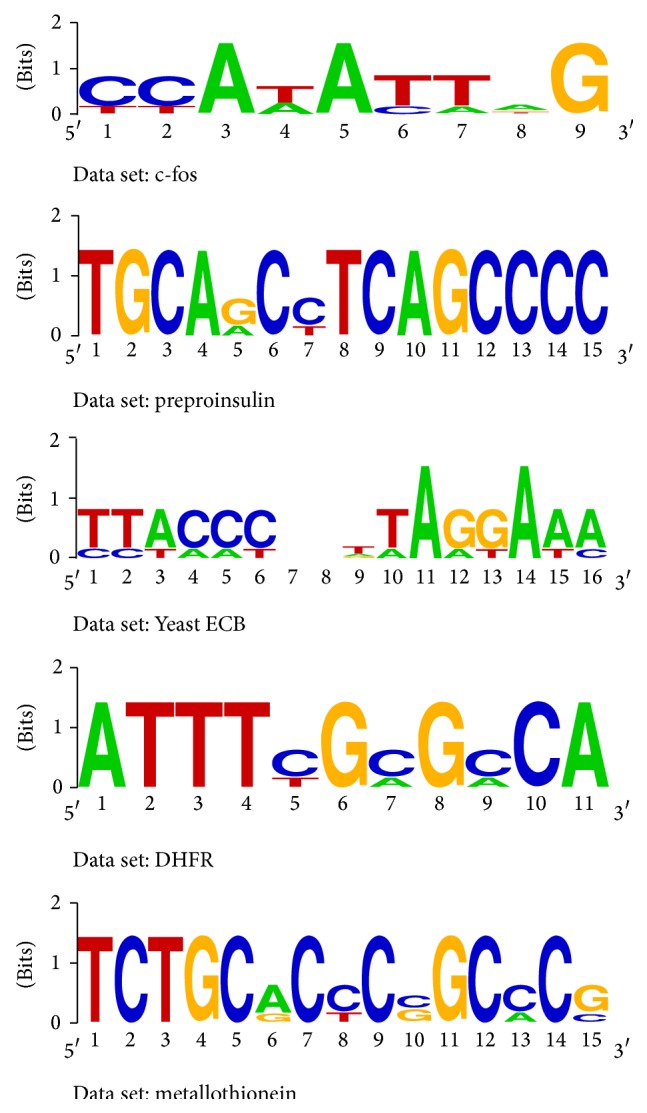
Sequence logos of the predicted motifs.

At first, the performance of APMotif is evaluated on the five widely used real data sets discussed in [21], which are preproinsulin, dihydrofolate reductase (DHFR), c-fos, metallothionein, and Yeast ECB. Because no information about the* l* and the *d* of the true motif is known in advance, we select the (*l*, *d*) used for each real data set as follows: the value of* l* is fixed as the length of the reference motif; the value of *d* is minimum to ensure that the predicted (*l*, *d*) instance contains the reference motif.  Table 4 shows that the predicted motifs returned by the APMotif algorithm are almost consistent with the reference motifs. In Figure 2, the software Weblogo [32] is used to show the sequence logos of the predicted motifs, which graphically shows the degree of motif conservation measured by relative entropy.

**Figure 3:**
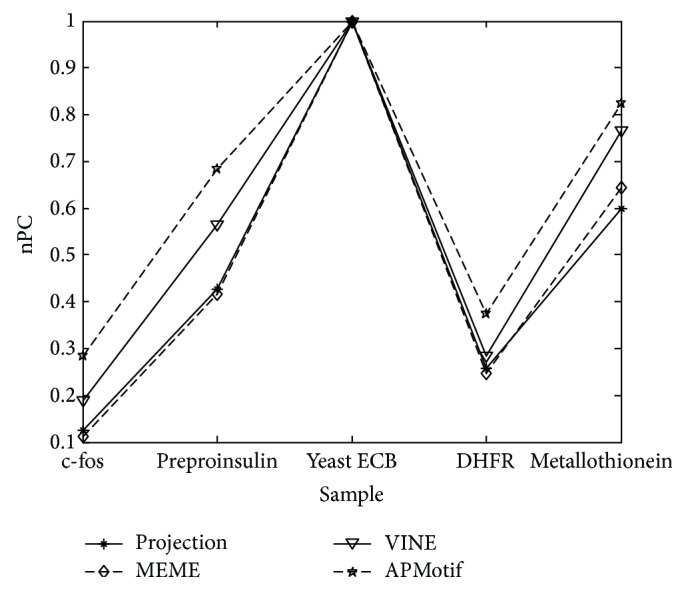
Prediction accuracy on real biological data.

For the five real data sets, Figure 3 compares the nucleotide level performance coefficient of APMotif with that of other popular algorithms. For Yeast ECB, the nPC of APMotif, MEME, Projection, and VINE are 1, which indicates the prediction result is completely correct. For c-fos, preproinsulin, DHFR, and metallothionein, the nPC of APMotif is 0.28, 0.68, 0.37, and 0.82, respectively, greater than that of three other widely used motif finding algorithms.

In addition, we show the prediction performance of APMotif on Tompa data [33], which is set up as the benchmark for testing motif discovery algorithms. For most Tompa data, the distribution of motif in each sequence makes it difficult to report the motif occurrence positions. We select some Tompa data. When a sequence contains more than one motif, it is difficult to discover all the motifs. When some sequences do not contain any motif, it is difficult to discovery motifs in other sequences. Overall, most algorithms have very low prediction accuracy in Tompa data. To improve the prediction accuracy, different algorithms should be executed together to complement each other.

**Figure 4:**
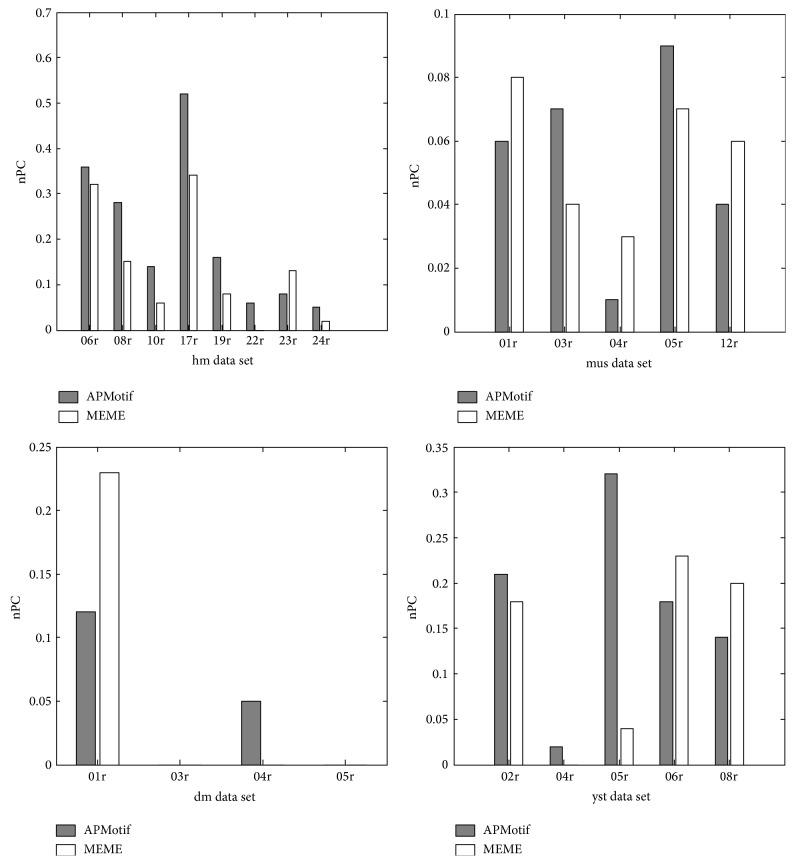
Prediction accuracy on Tompa data.

Figure 4 shows the prediction accuracy (nPC) of APMotif and MEME on each selected Tompa data. We observe that, for some data, such as hm08r, hm19r, mus03r, dm04r, and yst02r, the nPC of APMotif is better than that of MEME, but for some other data, such as hm23r, mus04r, dm01r, and yst06r, the nPC of MEME is better than that of APMotif. This phenomenon illustrates the practical significance in combining the results of APMotif and MEME to improve the ability of motif discovery algorithms in identifying TFBSs in higher eukaryotes [33].

## 4. Conclusions

The planted (*l*, *d*) motif search (PMS) problem arises from the need to find transcription factor binding sites (TFBSs) in DNA sequences. In this paper, we propose a new approximate algorithm, APMotif, which overcomes the local maximum drawback to some extent that is inherent in the EM motif-finding algorithms and guarantees that most motifs can be discovered for specific (*l*, *d*) settings. APMotif first constructs clusters by computing the Hamming distance between each* l*-mer in the reference sequence and all the* l*-mers in other sequences, and then it uses AP clustering combined with two metrics to select the high conserved clusters for the EM refinement progress. After the EM refinement, the cluster with maximum information content is verified as the motif instances. The experimental results on the synthetic data sets show that APMotif indeed removes most useless background information to obtain motifs with high accuracy. The experimental results on the real biological data show that APMotif can discover all or a large part of the motif instances. In summary, the APMotif algorithm outperforms the compared algorithms with significant improvement in prediction accuracy.

In the last years, the introduction of ChIP-Seq data raises new challenges for motif discovery problem from the perspective of data scale. Most existing motif discovery algorithms proposed for small data set are inefficient in dealing with ChIP-Seq data. Since APMotif performs AP clustering for multiple times with each clustering independent of others, APMotif thus features the merit for parallel computing. In our future work, we plan to parallel the APMotif algorithm to be fastened, so that the improved APMotif algorithm can deal with large data set efficiently.

## Figures and Tables

**Algorithm 1:**
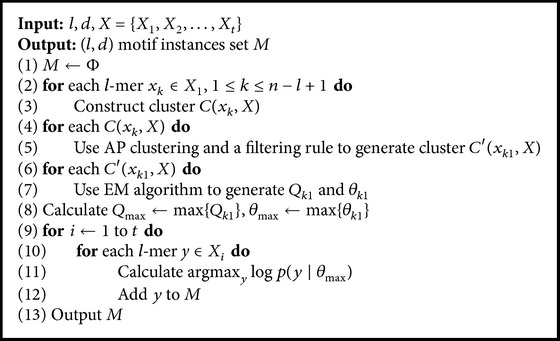
APMotif.

**Table 1:** Number of −*∞* related to different sequence length *n* on (15, 4) instance.

*n*	Data size^a^	Numbers of −*∞*	Percentage
100	93	6.81*e* + 03	78.75%
300	308	8.01*e* + 04	84.43%
500	523	2.29*e* + 05	83.89%
600	630	3.41*e* + 05	85.82%
800	846	5.84*e* + 05	81.55%
1000	1061	9.53*e* + 05	84.67%

^a^Data size: the number of all *l*-mers in one cluster.

**Table 2:** Prediction accuracy on different (*l*, *d*) instances.

(*l*, *d*)	nPC				
	Projection	MEME	VINE	Gibbs sampling	APMotif
(11, 3)	92%	65%	95%	56%	96%
(12, 3)	77%	84%	92%	3%	93%
(15, 4)	93%	86%	98%	19%	96%
(16, 5)	64%	71%	95%	2%	94%
(18, 6)	75%	79%	93%	3%	98%
(19, 7)	84%	77%	92%	4%	97%

**Table 3:** Prediction accuracy of different sequence length *n* on (15, 4) instance.

*n*	nPC				
	Projection	MEME	VINE	Gibbs sampling	APMotif
100	96%	99%	99%	92%	100%
300	94%	98%	99%	58%	99%
600	89%	91%	97%	19%	98%
800	87%	90%	98%	14%	97%
1000	88%	76%	91%	8%	95%

**Table 4:** Results of APMotif on real biological data.

Data set	Predicted motif	Reference motif	(*l*, *d*)
c-fos	CCATATTAG	CCANATTNG	(9, 2)
Preproinsulin	TGCAGCCTCAGCCCC	CAGCCTCAGCCCCAT	(15, 2)
Yeast ECB	TTACCCNNTTAGGAAA	TTTCCCNNTNAGGAAA	(16, 3)
DHFR	ATTTCGCGCCA	ATTTCGCGCCA	(11, 2)
Metallothionein	TCTGCACCCGGCCCG	CTCTGACNCCGCCC	(15, 2)
